# Herpes Zoster-induced Progressive Heart Block: A Case Report

**DOI:** 10.7759/cureus.3674

**Published:** 2018-12-03

**Authors:** Vahid Kazemi, Marlena C Fernandez, Kenneth Zide

**Affiliations:** 1 Internal Medicine, Aventura Hospital and Medical Center, Aventura , USA; 2 Internal Medicine, Aventura Hospital and Medical Center, Aventura, USA; 3 Cardiology, Aventura Hospital and Medical Center, Aventura, USA

**Keywords:** ekg, bradycardia, varicella zoster virus, heart block, cardiology, case report

## Abstract

Varicella-zoster virus (VZV) can produce painful, cutaneous lesions in human beings. Reactivation of this neurotropic virus leads to herpes zoster or shingles: a painful, unilateral, vesicular eruption in a restricted dermatomal distribution. Rarely, reactivation of this virus can lead to cardiac complications, such as myocarditis and even conduction abnormalities. In this case report, we present a patient with a cardiac complication post VZV reactivation and address an unusual question and concern resulting from latent VZV presentation in adults.

## Introduction

Varicella-zoster virus (VZV) is a DNA core virus with the ability to produce cutaneous lesions in human beings [1]. Clinical resolution of primary VZV infection is followed by the establishment of latent infection within the sensory dorsal root ganglia. Reactivation of this neurotropic virus leads to herpes zoster or shingles: a painful, unilateral, vesicular eruption in a restricted dermatomal distribution [2,3]​​​​​​. The incidence of herpes zoster increases when cell-mediated immunity against varicella zoster virus decreases due to aging or immunosuppressive disorders [4]. In rare and isolated cases, cardiac complications have been associated with the reactivation of VZV, such as myocarditis and conduction abnormalities. We present a case of a cardiac complication post VZV reactivation and address the question and concern of VZV latent presentation in adults.

## Case presentation

**Figure 1:**
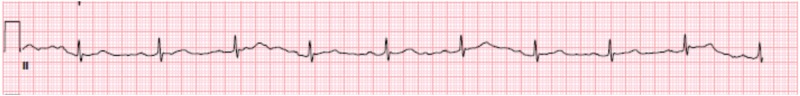
(3/15/18, 10:30 am) Sinus Bradycardia with 1st Degree AV Block. AV: Atrioventricular

A 64-year-old female with past medical history of diabetes mellitus presented to the emergency department in November 2017 for left eye pain of two days duration followed by left-sided neck rash. She complained of intermittent, shooting, burning pain along the distribution of this rash. She was examined and found to have nonconfluent grouped vesicles on the posterior neck and diagnosed clinically with herpes zoster reactivation (shingles). Initial electrocardiography (EKG) was unremarkable (no conduction blocks or evidence of pericarditis). She was treated with valacyclovir and oral steroids and advised to seek urgent ophthalmological evaluation at a specialized center. She was readmitted in March of 2018 after presenting with altered mental status. EKG performed at that time showed a new first degree atrioventricular (AV) block as shown in Figure 1 below.

**Figure 2:**
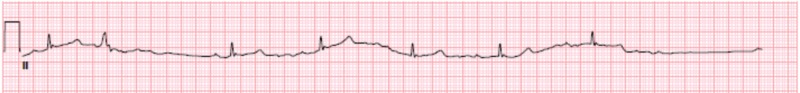
(3/15/18, 18:07) Sinus Rhythm with 2nd Degree AV Block (Mobitz I). AV: Atrioventricular

Admission vitals were blood pressure of 96/58, heart rate of 65 beats per minute. Laboratory studies done on admission were unremarkable except for hypoglycemia at 32. Later on that day, she developed bradycardia at 49 beats per minute with blood pressure 117/63 mm Hg. Repeat EKG was performed, this time showing Second Degree AV block (Mobitz I) as seen in Figure 2.

**Figure 3:**
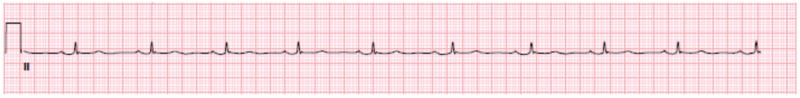
(3/16/18, 21:22) Sinus Bradycardia with 1st Degree AV Block. AV: Atrioventricular

**Figure 4:**
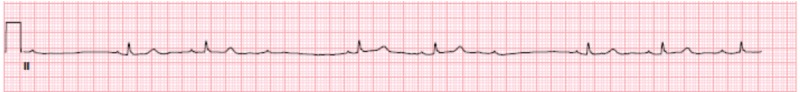
(3/18/18, 14:25) Sinus Rhythm with 2nd Degree AV Block (Mobitz I). AV: Atrioventricular

She denied any fever, chills, arthralgias, myalgias, vomiting or diarrhea. She also denied any chest pain, palpitations, or shortness of breath. Electrolyte abnormalities were corrected and she was continuously monitored on telemetry and followed with serial EKGs (Figures 3-4).

She had a positive chronotropic response with exertion and awakening. Echocardiography done during admission showed preserved left ventricular function (60%) with no wall motion abnormalities and mild grade 1 diastolic dysfunction. Electrophysiology evaluation was obtained, who advised outpatient monitoring within two weeks to evaluate for progression. The patient was not felt to be at high risk of progression due to preservation of the chronotropic response as well as absence of high-grade block.

## Discussion

VZV was first recognized as a possible cause of myocarditis in 1953 [5]​​​​​​. A case report in 1971 first demonstrated VZV myocarditis, specifically affecting the conducting system of the heart, in an infant with multiorgan involvement [6]. This led to the suggestion that VZV myocarditis could lead to cardiac conduction disturbances and supraventricular, later ventricular, arrhythmias have been documented in infants with varicella zoster [7]. Possible areas in which VZV may infect and lie dormant include the nodose and celiac (autonomic) ganglia as well as dorsal root ganglia. Ma et al. described a case of complete heart block in the setting of acute/recent VZV reactivation. An etiological link between the VZV infection and delayed heart block was suggested by a persistent elevation of anti-varicella zoster virus IgM antibodies. There was also a progressive and discernible slowing of the heart rate following the onset of zoster pain that culminated in bradycardia due to complete heart block [8]. Since no direct neural or vascular connections are present between dorsal root ganglia and the cardiac sympathetic/parasympathetic ganglia, a coincident varicella zoster virus reactivation may occur at both the dorsal root ganglion and the cardiac sympathetic ganglia. The preservation of chronotropic response in our patient is suggestive of a partial, rather than complete block of the cardiac sympathetic ganglion, or may simply reflect a weakening of resting sympathetic tone. Fortunately, there is evidence showing VZV-related conduction abnormalities may be reversible with proper management of the underlying condition [9]. In our case, the patient’s heart pathology and EKG changes started more than three months after the skin rash and VZV reactivation began. Although other viral serologies (e.g., coxsackie, HIV) were not obtained in our patient, the temporal relationship of zoster reactivation on multiple occasions with lymphocytosis, shortly followed by cardiac conduction abnormalities, leads us to believe that VZV reactivation may be responsible for these findings. If so, our patient’s progression from first degree heart block to second degree (Mobitz I) happened in less than six hours, which to our knowledge, is a highly atypical progression. The other notable finding was reversibility of the cardiac conduction disturbance, which started hours later.

## Conclusions

The possibility that a conduction disturbance linked to varicella zoster virus reactivation can progress in just a couple of hours or even days is a cause for concern. In our opinion, this warrants cardiac telemetry monitoring for any dangerous findings which may require urgent management should a patient be diagnosed with reactivated varicella. In the end, given the increasing incidence of VZV reactivation in adults the possible complications, especially significant cardiac conduction abnormalities, should be kept in the differentials when treating these patients.
